# Consistency of Mycobacterium tuberculosis Complex Spoligotyping between the Membrane-Based Method and *In Silico* Approach

**DOI:** 10.1128/spectrum.00223-22

**Published:** 2022-04-25

**Authors:** Charlotte Genestet, Elisabeth Hodille, Albin Bernard, Maxime Vallée, Gérard Lina, Adrien Le Meur, Guislaine Refrégier, Oana Dumitrescua

**Affiliations:** a Centre International de Recherche en Infectiologie (CIRI), Ecole Normale Supérieure de Lyon, Université Claude Bernard Lyon-1, Inserm, Lyon, France; b Hospices Civils de Lyon, Institut des Agents Infectieux, Laboratoire de Bactériologie, Lyon, France; c Centre national de la recherche scientifique (CNRS), AgroParisTech, Ecologie Systématique et Evolution, Université Paris-Saclay, Gif-sur-Yvette, France; University Paris-Saclay, AP-HP Hôpital Antoine Béclère, Service de Microbiologie, Institute for Integrative Biology of the Cell (I2BC), Commissariat à l′énergie atomique et aux énergies (CEA), CNRS

**Keywords:** tuberculosis, *Mycobacterium tuberculosis* complex, spoligotyping, membrane-based spoligotyping, *in silico* spoligotyping, whole-genome sequencing, CRISPR-builder-TB

## Abstract

To tackle the spread of tuberculosis (TB), epidemiological studies are undertaken worldwide to investigate TB transmission chains. Clustered regulatory interspaced short palindromic repeats (CRISPR) locus diversity, also called spoligotyping, is a widely used genotyping assay for the characterization of Mycobacterium tuberculosis complex (MTBC). We compared herein the spoligotyping of MTBC clinical isolates using a membrane-based method (following an initial PCR step) and whole-genome sequencing (WGS)-based method (i.e., *in silico* spoligotyping). All MTBC strains isolated at the Lyon University Hospital, France, between November 2016 and December 2020 were included (*n* = 597). Spoligotyping profiles were also used for species identification among the MTBC. Outputs of both methods were analyzed, and discrepant results were investigated thanks to CRISPRbuilder-TB. The overall agreement was 85.7%. Spacer discrepancies observed between the methods were due to the insertion of IS6110 within the direct repeat (DR) sequence upstream or downstream of spacers, mutated DR sequences, or truncated spacers. Discrepancies did not impact species identification. Although spoligotyping-based species identification was inconclusive for 29 isolates, SNP-based phylogeny conducted after WGS allowed the identification of 23 M. tuberculosis (Mtb), 2 M. canettii, and 4 mixed MTBC infections. WGS yielded very few discrepancies compared to membrane-based spoligotyping. Overall agreement was significantly improved (92.4%) by the CRISPR locus reconstruction using CRISPRbuilder-TB for the MTBC isolates with the shared international type 53 *in silico* spoligotyping. A smooth transition from the membrane-based to the *in silico*-based genotyping of M. tuberculosis isolates is, therefore, possible for TB diagnosis and epidemiologic survey.

**IMPORTANCE** Whole-genome sequencing (WGS) has profoundly transformed the perspectives of tuberculosis (TB) diagnosis, providing a better discriminatory power to determine relatedness between Mycobacterium tuberculosis complex (MTBC) isolates. Previous genotyping approaches, such as spoligotyping consisting of an initial PCR step followed by reverse dot hybridization, are currently being replaced by WGS. Several pipelines have been developed to extract a spoligotype from WGS data (*in silico* spoligotyping) allowing for the continuity of MTBC molecular surveys before and after WGS implementation. The present study found very good overall agreement between hybridization to membrane-based spoligotyping and *in silico* spoligotyping, indicating the possibility of a smooth transition from the traditional to the *in silico*-based genotyping of MTBC isolates for TB diagnosis and epidemiological survey.

## INTRODUCTION

Control of Mycobacterium tuberculosis complex (MTBC) transmission in high-income and low tuberculosis (TB) prevalence countries remains a public health priority given the constant changes in MTBC epidemiology worldwide. Key measures for TB control rely on the linkage of cases and identification of transmission chains, through a population-based systematic molecular TB survey, to uncover outbreaks, even between unrelated cases (1, 2).

Two major genotyping assays for MTBC isolates have been developed and employed across numerous epidemiological studies: the spacer oligonucleotide typing (spoligotyping) and the mycobacterial identification repetitive unit-variable number of tandem repeats 15 (MIRU-VNTR15) typing (3–5). Historically, spoligotyping detected the presence or absence of 43 unique spacers in the direct repeat (DR) region of the clustered regularly interspaced short palindromic repeats (CRISPR) locus of MTBC. This was based on an initial PCR using primers directed to the most frequently occurring DR sequences, called DR0, followed by a reverse line blot hybridization membrane-based revelation method (6, 7). More recently, and using whole-genome sequencing (WGS), 68 spacers for the MTBC were identified (98 including M. canettii) (8). Because the diversity of the CRISPR locus has been shown to accurately reflect the phylogeny of MTBC, spoligotyping has not solely been used for epidemiological purposes but also for MTBC species identification using an algorithmic approach in routine TB diagnosis (9).

Since the advent of next-generation sequencing (NGS), WGS has been implemented in high-income countries and profoundly transformed the perspectives of TB diagnosis. WGS provides a better discriminatory power than spoligotyping and MIRU-VNTR15-typing to determine relatedness between MTBC isolates (10). In addition, WGS allows obtaining quick and accurate genotypic antimicrobial susceptibility testing and MTBC species identification using single nucleotide polymorphism (SNP) calling without prior specific PCR amplification (11). Moreover, several pipelines have been developed to extract a spoligotype from WGS data, also called *in silico* spoligotyping (12), enabling continuity of MTBC molecular surveys. Nevertheless, to ensure a smooth transition from the hybridization method to the *in silico*-based assay, the consistency of both outputs needs to be thoroughly investigated. It is well known that the initial amplification step of the former (that is not required for the latter) may introduce bias in the output sequences mainly due to heterogeneous or variant-sensitive primer affinity (13). In a recent study, Bogaerts et al. (14) compared these methods using 166 MTBC from the Belgian National Reference Center, but the discrepancies were not explored. In the present study, we compared WGS-based MTBC identification and spoligotyping (43 spacers) with those obtained by membrane hybridization assays in a French cohort. The discrepancies were further analyzed using CRISPRbuilder-TB, allowing a reconstruction of the MTBC CRISPR locus to understand the underlying causes.

## RESULTS

### Comparison between MTBC species identification using membrane-based spoligotyping and WGS SNP calling.

A total of 597 MTBC isolates were analyzed, among which there were 4 cases of mixed infections (infection with 2 different strains of MTBC), not allowing species or lineage identification using spoligotyping. For the 593 remaining MTBC isolates, identification of MTBC species using membrane-based spoligotyping was feasible for 568 isolates (95.8%). For 25 MTBC isolates, spoligotyping did not allow species identification, including 21 “unknown” spoligotypes, i.e., spoligotyping was not related to an MTBC species in the SITVIT database, and 4 isolates with the shared international type (SIT) 2669 for which none of the 43 spacers was detected.

**FIG 1:**
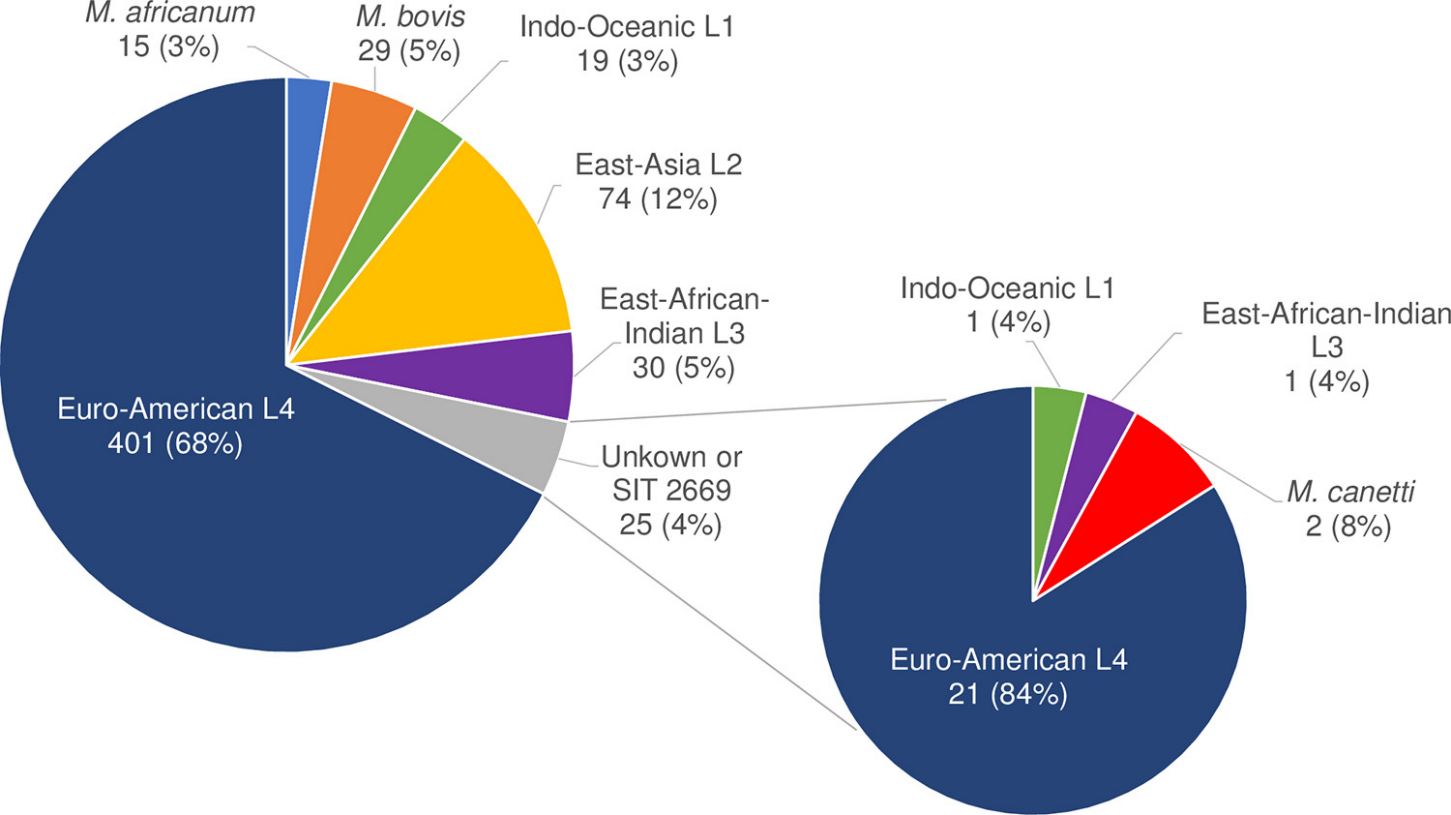
Identification of 593 MTBC isolates. Sector diagram on the left: membrane-based spoligotyping identification. Sector diagram on the right: whole-genome sequencing single nucleotide polymorphism calling identification for MTBC isolates classified as “Unknown” or standard international type (SIT) 2669 by spoligotyping identification.

For these 568 isolates identified by membrane-based spoligotyping, all identifications were concordant with MTBC species found by WGS SNP calling. The majority were M. tuberculosis (*n* = 524, 92.3%), including 401 Euro-American lineage 4, 74 East-Asian lineage 2, 30 East African-Indian lineage 3, and 19 Indo-oceanic lineage 1, 29 M. bovis, and 15 M. africanum (Fig. 1). WGS SNP calling allowed MTBC species identification for the 25 MTBC isolates not identified by spoligotyping. Among these, 2 were M. canettii (spoligotyping 2669 for which no spacer was detected) and 23 were M. tuberculosis, including 21 Euro-American lineage 4 (including the other two SIT 2669), 1 East African-Indian lineage 3, and 1 Indo-oceanic lineage 1 (Fig. 1).

For the 4 cases of mixed MTBC infection, only WGS SNP calling allowed the accurate identification of the 2 MTBC isolates contained in the samples. In 3 cases, mixed infection was with the M. tuberculosis L2-Beijing strain and an M. tuberculosis L4-Euro-American strain, and in 1 case the mixed infection was with an M. tuberculosis L4-Euro-American strain and an M. tuberculosis L3-East African-Indian strain.

### Concordance between membrane-based spoligotyping and *in silico* spoligotyping.

Excluding mixed infections, the overall agreement between membrane-based spoligotyping and *in silico* spoligotyping at the sample level was 85.7% (508/593; 95% confidence interval, 95%CI [82.6, 88.4], Table S1). Of the 85 isolates with discordant MTBC spoligotypes, 75 differed by only 1 spacer, and 10 isolates differed by 2 spacers.

At the spacer level, the most discordant was spacer 31, which concerned 61 isolates (71.8%). It was always absent in the membrane-based method but present in *in silico* spoligotyping (Table 1).

**FIG 2:**
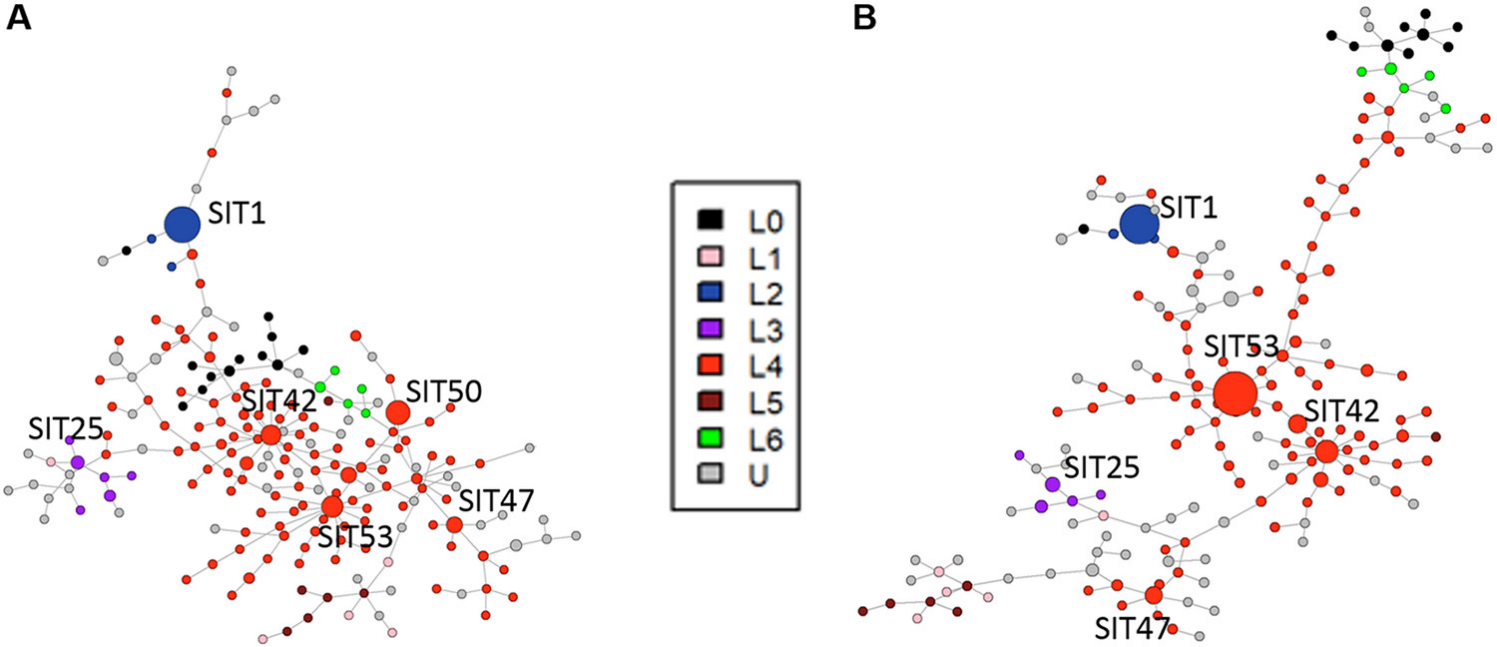
Minimum spanning trees of the test sample featuring spoligotype diversity as studied either using membrane-based spoligotyping or *in silico* WGS-based spoligotyping. (A) Membrane-based spoligotyping. (B) WGS-based spoligotyping. Nodes are colored according to the corresponding Lineage (see legend). L0: animal-adapted lineages, such as M. bovis; U: unknown. Node size corresponds to the population size with the corresponding spoligotype. Most prevalent patterns (*n* >7) are labeled with their standard international type (SIT).

Among these discrepancies involving spacer 31, 39/61 (63.9%) were found in MTBC isolates with the SIT 50 on membrane-based spoligotyping and SIT 53 on *in silico* spoligotyping. Of note, in the study data set the SIT 50 was never obtained with *in silico* spoligotyping: all SIT 50 on membrane-based spoligotyping were SIT 53 on *in silico* spoligotyping (Table S1, Fig. 2). Except for spacer 31, Cohen’s kappa indicated that the concordance between *in silico* and membrane-based spoligotyping indicated an almost perfect agreement at the spacer level (Cohen’s kappa > 0.81; Table 1).

**TABLE 1:** Discordant spacers between membrane-based spoligotyping and *in silico* spoligotyping

Presence or absence^*a*^	Discordant spacer	Prevalence in membrane-based spoligotyping, n	No. of concerned isolates	Overall agreement, %	Cohen’s kappa
“0” in membrane, “1” *in silico*	6	447	1	99.8	0.99
	10	419	4	99.3	0.98
	11	440	2	99.7	0.99
	14	444	5	99.2	0.98
	15	441	7	98.8	0.97
	20	444	3	99.5	0.99
	26	389	4	99.3	0.99
	31	320	61	89.7	0.79
	32	429	2	99.7	0.99
	38	544	1	99.8	0.99
	39	515	1	99.8	0.99
	42	522	2	99.7	0.98
“1” in membrane, “0” *in silico*	18	447	1	99.8	0.99

a “0” meaning the absence of the spacer; “1” meaning the presence of the spacer.

### Discrepancy analysis.

To better understand the discrepancies observed between membrane-based spoligotyping and *in silico* spoligotyping, the 85 discrepant spoligotypes were analyzed using CRISPRbuilder-TB, allowing a reconstruction of the CRISPR locus in MTBC (8). This found an insertion of the mobile element IS6110 at 5 nucleotides from the end of the DR sequence upstream of the spacers 6, 10, 11, 15, 20, 26, 31, or 32. An insertion of IS6110 at 6 nucleotides from the start of the DR sequence downstream of the spacer 31 was also found and was responsible for all conversions of SIT 50 according to membrane-based spoligotyping to SIT 53 according to *in silico* spoligotyping. The CRISPRbuilder-TB analysis found mutated DR sequences leading to DR other than DR0 for some of the discrepancies observed for the spacer 15 (DRb2 downstream of the spacer) and those observed for the spacer 42 (DR6 downstream of the spacer). It also found truncated spacers for the discrepant spacer 38 and some of the discrepancies observed for the spacers 14 and 15. Analysis of the 4 SIT 2669 for which no spacer of the 43 investigated by the conventional spoligotyping methods were detected using CRISPRbuilder-TB found that 2 M. canettii isolates had spacers of the 98-spacer spoligotyping format reported by Guyeux et al. (8). For the 2 M. tuberculosis isolates SIT 2669, DR/CRISPR regions were completely deleted. CRISPRbuilder-TB did not find any event for the discrepancies observed for the spacers 18 and 39.

## DISCUSSION

The MTBC CRISPR locus is the preferential insertion site for the IS6110, possibly disrupting DR or adjacent spacer sequences (15). Both DR variations and IS insertion may hamper primer affinity resulting in incomplete or abortive DNA amplification, thus changing expected spoligotype patterns, despite the presence of spacers in the CRISPR locus (16–18). Thus, as observed in the present study, previous studies not using WGS reported that the insertion of IS6110 around the spacer 31 led to erroneous spoligotypes with the conventional PCR first-step methods (16–18), but, unlike herein, they did not report this for spacers 6, 10, 11, 15, 20, 26, and 32. These insertions of IS6110 within the DR sequence upstream or downstream of spacers probably lead to an asymmetrical split of the primer targets and a failure to detect the spacers in conventional spoligotyping methods using an initial PCR step. The presence of other genetic alterations of the CRISPR locus also explained the failure to detect the spacers by the conventional PCR first-step membrane-based spoligotyping methods such as mutations of the DR sequence (that is, likely to reduce the affinity of the primers; spacer 15 and 42), and the truncation of a spacer (that is, likely to prevent the detection by hybridization; spacers 14, 15, and 38).

The overall agreement between membrane-based and *in silico* spoligotyping at the sample level was 85.7% (508/593; 95% CI [82.8, 88.5] despite the discrepancies due to genetic variations described above. Recently, Bogaerts et al. found a similar overall agreement (89.2%) on a set of 166 MTBC isolates (14). As observed herein, the spacer 31 concerned the most mismatched spacer, and in all cases of mismatch, it was detected using *in silico* spoligotyping and not the traditional spoligotyping method. Although most of these discrepancies were strains identified by membrane-based spoligotyping as SIT 50 that were identified by *in silico* spoligotyping as SIT 53, these followed a distinct evolutionary pathway marked by the insertion of IS6110 within the DR sequence downstream the spacer 31 (6, 19, 20). Due to the phylogenetic relevance of this insertion, the classification of the corresponding strains as SIT 50 instead of SIT 53 should be preferred. Thus, to prevent misclassification, for strains assigned to SIT 53 by *in silico* spoligotyping, analysis of WGS data using CRISPRbuilder-TB may restore the SIT 50 pattern for those harboring the IS6110 insertion within the DR sequence downstream of the spacer 31. This approach would have significantly improved the overall agreement between membrane-based spoligotyping and *in silico* spoligotyping herein from 85.7% (508/593; 95%CI [82.6, 88.4]) to 92.4% (547/593; 95%CI [89.9, 94.4], *P* = 0.0003; unpublished data).

The present study found that the MTBC strains lacking all 43 spacers investigated by the conventional spoligotyping method and assigned to the SIT 2669 were extremely diverse according to the WGS. Some strains were identified as M. canetti, and the others that had a completely deleted DR/CRISPR region belonged to genetically unrelated L4-EuroAmerican M. tuberculosis lineage as previously described for the SIT 2669 isolates (21). These observations highlight that the SIT 2669 was not meaningful for both epidemiological studies and rapid species identification. In contrast, for the MTBC isolates tested herein with SITs other than 2669, there was a complete overall agreement for species identification by WGS SNP calling or membrane-based spoligotyping, indicating the relevance of both methods for the species identification. Nevertheless, species identification based on SNP calling from WGS presented two advantages over species assignement by spoligotyping. First, WGS SNP calling allowed the identification of 25 MTBC isolates for which species assignment by spoligotype-based algorithms was inconclusive, including 4 strains of the SIT 2669 for which no spacer was amplified. Second, only WGS allowed accurate identification of the 4 mixed infections whereas a false membrane spoligotype pattern resulted from the superimposed profiles of the mixed strains.

The overall agreement was further improved to 92.4% (547/593; 95%CI [89.9, 94.4]) by supplementary analysis using CRISPRbuilder-TB for certain isolates. In addition to more accurate epidemiological monitoring than that provided by spoligotyping, WGS had an added value in some cases of species identification. These data support a smooth transition from the membrane-based to the *in silico*-based genotyping of M. tuberculosis isolates is therefore possible for TB diagnosis and epidemiologic survey.

## MATERIALS AND METHODS

### MTBC isolates.

Between November 2016 and December 2020, MTBC isolated from specimens taken from patients during routine care in the Lyon University Hospital, France were prospectively included.

All data were maintained in an electronic database, in accordance with the ethics committee of the Lyon university hospital, France (Comité d'Éthique du CHU de Lyon, number: 20-216), and the national data protection commission (Commission nationale de l'informatique et des libertés; reference methodology MR-004 that covered the processing of personal data for purposes of study, evaluation or research that did not involve the individual). In accordance with French legislation, written informed consent from patients was not required.

### MTBC conventional spoligotyping.

Membrane-based spoligotyping was performed as described elsewhere (6). MTBC spoligotyping-based identification and SIT number determination were provided through the open-access SITVITWEB (22) and SpolLineages software tool (https://github.com/dcouvin/SpolLineages) (9).

### MTBC WGS.

For MTBC WGS, genomic DNA was purified from cleared lysates using the Maxwell RSC Instrument (Promega, Madison, WI, USA) automated DNA extraction system and the Maxwell RSC Blood DNA kit (Promega). Libraries were generated using a bead-based tagmentation system (DNAprep; Illumina, San Diego, CA, USA). A nanoliter liquid handler (mosquito HV; SPTLabtech, Hertfordshire, UK) was used to reduce the by 10 times the reaction volumes. Miniaturized libraries were sequenced on the Nextseq or Miseq system (Illumina) to produce 150 or 300 base-pair paired-end reads, respectively. Reference genome coverage was at least 96% and depth of coverage at least 30×.

### MTBC *in silico* spoligotyping.

MTBC *in silico* spoligotyping was determined through the open-access tool SpoTyping (https://github.com/xiaeryu/SpoTyping-v2.0.) (12). MTBC spoligotyping-based identification and SIT number determination were provided as described above. Moreover, discrepancies between membrane-based spoligotyping and *in silico* spoligotyping were determined using CRISPRbuilder-TB (https://github.com/cguyeux/CRISPRbuilder-TB) (8) to identify events responsible for a spacer found absent “0” in membrane-based but present “1” in *in silico* spoligotyping or vice versa.

### MTBC WGS SNP calling identification.

The metrics relating to the quality of raw WGS reads were measured using FastQC (https://www.bioinformatics.babraham.ac.uk/projects/fastqc/), and potential cross-species contaminations were monitored by FastQ Screen (23). Mapping quality controls were performed using Samtools stats (24). All reports in a sequencing run were compiled using MultiQC (25). Quality control samples then underwent mapping on the Mycobacterium tuberculosis reference genome (NC_000962.3) using Burrow-Wheeler Aligner (BWA) (https://github.com/lh3/bwa) (26). Following the 2021 guidelines from the Genome Analysis Toolkit (GATK) (https://github.com/broadinstitute/GATK-for-Microbes), samples were aligned both on reference and on shifted reference. Duplicated reads were identified using Picard MarkDuplicates (http://broadinstitute.github.io/picard/). Variant calling with MuTect2 in microbial mode was performed (27).

Finally, variant calls were processed using the open-access SNP-IT tool (https://github.com/samlipworth/snpit) (28) to identify MTBC lineage based on WGS SNP calling (29).

### Data analysis.

Cohen’s Kappa values were calculated using XLSTAT 2020.5.1 (Addinsoft, Paris, France), and interpreted according to Landis and Koch criteria (30). Minimum spanning trees were built using Rstudio: pairwise distances were computed using Manhattan metrics, and the graph was built using the igraph tool in R (31).
